# P-592. Rapid Response to the First Locally Acquired Dengue Infections in California: Dengue Serosurveillance in Southern California

**DOI:** 10.1093/ofid/ofaf695.806

**Published:** 2026-01-11

**Authors:** Sara Y Tartof, Gregg S Davis, Christopher N Mores, Jessica Skela, Vennis Hong, Rudy A Patrick, Magdalena E Pomichowski, Iris Anne C Reyes, Madeline Kernahan, Jonathan P Bashor, Adrienne Mackenzie, Michael Aragones, Alec D Gilfillan, Banshri Kapadia, Adrienne Epstein, Hui Zhou, Isabel Rodriquez-Barraquer, Joseph Lewnard

**Affiliations:** Kaiser Permanente Southern California, Pasedena, CA; Kaiser Permanente Southern California, Pasedena, CA; The George Washington University, Washington, District of Columbia; Kaiser Permanente Southern California, Pasedena, CA; Kaiser Permanente Southern California, Pasedena, CA; Kaiser Permanente Southern California, Pasedena, CA; Kaiser Permanente Southern California, Pasedena, CA; Kaiser Permanente Southern California, Pasedena, CA; The George Washington University, Washington, District of Columbia; The George Washington University, Washington, District of Columbia; The George Washington University, Washington, District of Columbia; Kaiser Permanente Southern California, Pasedena, CA; Kaiser Permanente Southern California, Pasedena, CA; Kaiser Permanente Southern California, Pasedena, CA; University of California, San Francisco, San Francisco, California; Kaiser Permanente Southern California, Pasedena, CA; University of California, San Francisco, San Francisco, California; University of California, Berkeley, Berkeley, California

## Abstract

**Background:**

In October 2023, the first locally acquired dengue case was reported in California; 20 cases have since been reported. Due to asymptomatic infections and sporadic testing, the true burden of dengue virus (DENV) in California is likely much larger. We rapidly launched widespread serosurveillance in Southern California following the first detected case to monitor infections and characterize background population immunity.

**Figure:**
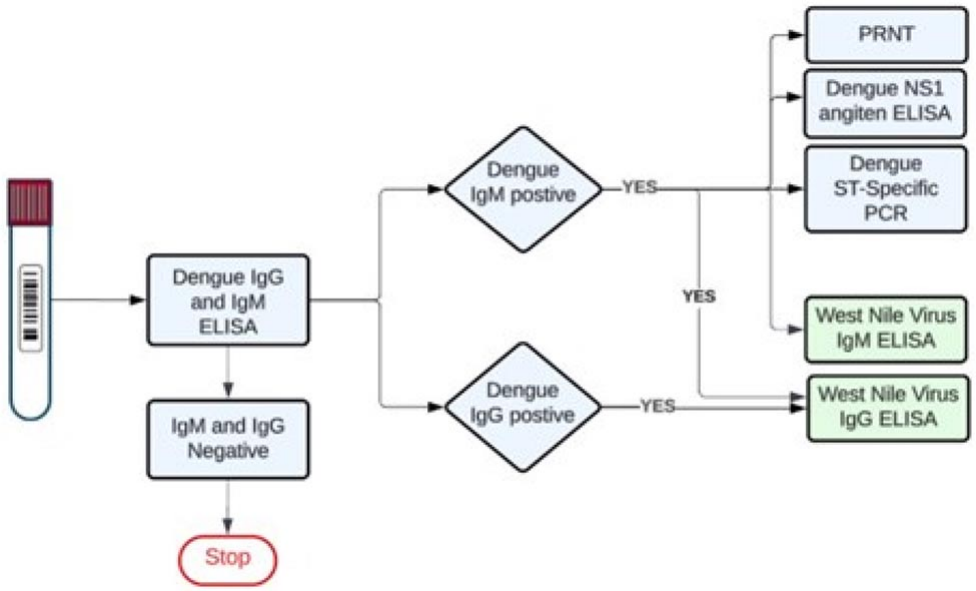
Serum Specimen Testing Workflow

**Figure:**
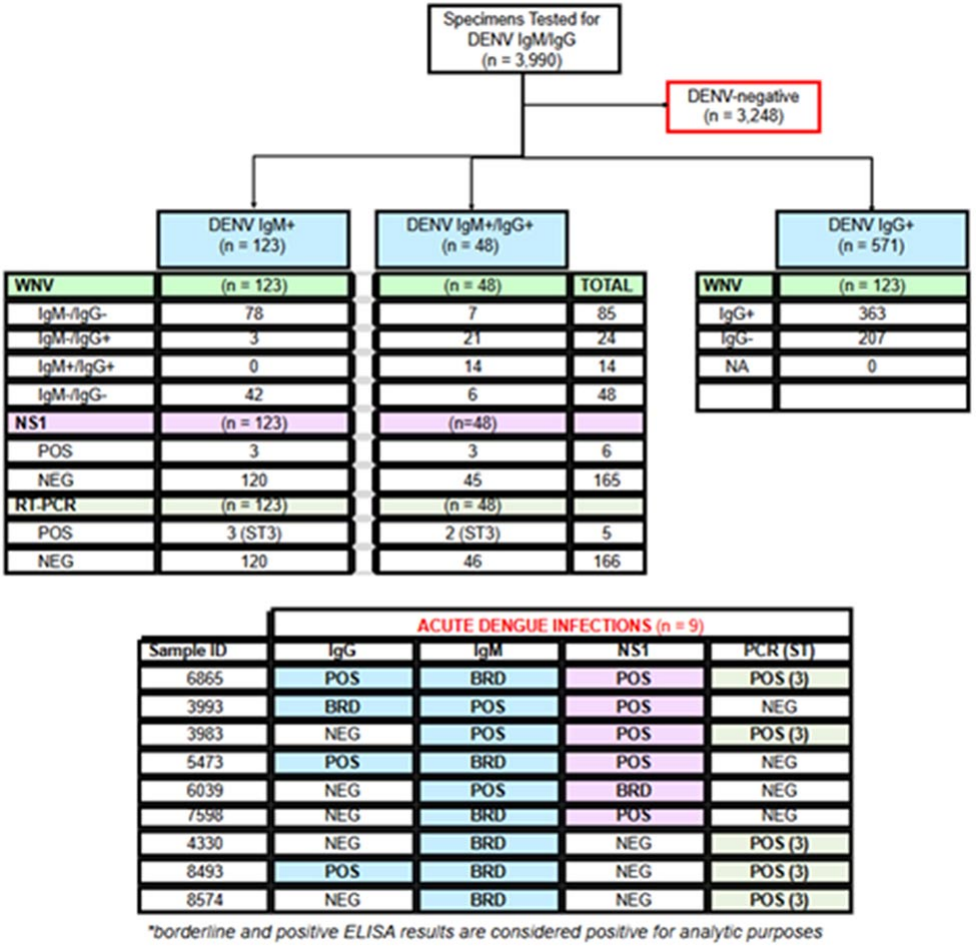
Dengue and West Nile Virus Testing Results

**Methods:**

In November 2023, we initiated remnant serum specimen collection from Kaiser Permanente Southern California members presenting with febrile illness ≤7 days of standard-of-care serum specimen collection, and/or residing within the same or neighboring ZIP code as a locally acquired case. Select specimens were tested by ELISA for IgM antibodies against DENV (marker of recent infection) and IgG (marker of historical infection). All DENV IgM-positive samples were additionally tested for dengue NS1 antigen and by serotype-specific dengue RT-PCR (Figure 1). To further characterize potentially cross-reactive antibody responses, DENV IgG or IgM-positive specimens were tested for West Nile Virus (WNV) IgM and IgG antibodies. Plaque reduction neutralization assays are ongoing.

**Results:**

Of 15,521remnant serum specimens, 3,990 specimens were tested by ELISA for DENV IgM and IgG antibodies. Among these, 14.3% (571) specimens were DENV IgG-positive, 3.1% (123) were DENV IgM-positive, and 1.2% (48) DENV IgM/IgG positive. Of the 171 IgM-positive specimens, 9 were either NS1 or DENV PCR positive (4 NS1 antigen-positive, 3 DENV-3 PCR positive, 2 NS1/DENV-3- PCR positive). DENV 1, 2 or 4 were not detected by PCR among the IgM-positive specimens. WNV antibodies were detected in 64% (363/571), 37% (45/123), and 85% (41/48) of the DENV IgG-, IgM- and IgG/IgM-positive specimens, respectively (Figure 2).

**Conclusion:**

These data represent the first estimates of population immunity to DENV in Southern California and suggest underreporting of DENV infections during recent years. These results will inform estimation of attack rates and the ratio of reported to true infections. These data also serve as a baseline to monitor changes in population seroprevalence to DENV over coming years, and the size of the population at risk for severe dengue disease due to prior infection.

**Disclosures:**

Sara Y. Tartof, PhD, MPH, Centers for Disease Control and Prevention: Grant/Research Support

